# Supplementary material for: Identification of the WUSCHEL-Related Homeobox (WOX) Gene Family, and Interaction and Functional Analysis of TaWOX9 and TaWUS in Wheat
Source: Int J Mol Sci. 2020 Feb 26;21(5):1581. doi: 10.3390/ijms21051581 (PMC7084607; doi:10.3390/ijms21051581)
Supplement: Supplementary file 1 [file ijms-21-01581-s001.zip › Supplementary Table S3.docx]

**Supplementary Table S3:** The full length coding sequences (CDS) used for the subcellular localization and BiFC assay

| **Gene Name** | **Complete CDS** |
| --- | --- |
| **TaWUS** | ATGGACAAGCAGAGCGTCATGTGGAGACAGCTGCAGCACCAGCACCACCAGGCCAGCGGCGCGGCGGGTGGCAATGCCGTCGCTGCTGCCGGTGCTGGTGCGACGACGACGACCGCGCCCGTCCGGCCGAGCGGCGCGCGGTGGACGCCCACGCCGGAGCAGGTCAAGATCCTCAAGGACCTCTACTACGACTGCGGCATCCGGTCGCCCACGGCGGAGCAGATCCAGCGGATCGCGGCCCGGCTCCGGCAGTACGGCCGGATCGAGGGCAAGAACGTCTTCTACTGGTTCCAGAACCACAAGGCCCGCGAGCGCCAGAAGAAGCGCCTCGGCGTCGACGTCAACGGCTCCCCCCTCGCCACCGCCACCGCCGCCGACGTCCTCGCCCTCTCCCCCTCCGGTGCGGCGGCCGGCTTGTACGGCGCCGGCAGCTGCGCCGGTAGAGGCGCGGCTGTCCATCCAGATGCGAGCGCCACTACTACTACTTGCTGGGGAGACAGCACCCTGCAGGACTACATGGGCGCGAGGAGCACGGCGGGAGCGGGCAACCACGGCGGCGCCGCCGCAGCAGCACCTACGCCGTGGCCAGCTGCGAGCTTCCCTTTCTCCACCAACCAGACGCCGCCAATGCCCCCGCCGCGGGAGCTCCCGCTCTTCCCGACCGGCGGAGGCCGGCAAGAAAGCGCCGACGACTTCAACGGCAGCAGCTACCATCTCCAGCCCAACAGCTCGCAGTGGTGGGAAGCTGCCGCCGCCAGCAACGCCAACGCCATGGCAGTCGTCCATCATCAGCTGCTGCAAGAACAGCACGAGCAGCAGCACAGCTTTTACAACAGCAGCGGCAACCAGCAGCAGATGATGATGATGATGCCCATCCAGGACGCCGGCACCTCCCCGGAGCTCACTCTCCGCGCCCCTTACATGTGA |
| **TaWOX9** | ATGGAGGCGCTGAGCGGGCGGGTGGGAGTGAAGTGCGGGCGGTGGAACCCGACGGCGGAGCAGGTGAAGGTGCTGACGGAGCTGTTCCGGGCGGGGCTGCGGACGCCGAGCACGGAGCAGATCCAGCGGATCTCCACCCACCTCAGCGCCTTCGGCAAGGTGGAGAGCAAGAACGTCTTCTACTGGTTCCAGAACCACAAGGCCCGCGAGCGCCACCACCACAAGAAGCGCCGCCGCGTCGCCTCCTGCTCCCCCGACAGCAGCAGCAACGAGGAAGAGACCGGCCGTGCTGCTGGCCGCGACGCCGAGCCCGCCGACCTCGTGCTCCAGCCACCCGAGAGCAAGCGTGAGGCCAGAGGCTACAACCACCACCCCCGGATCATGACATGCTATGTGAGGGAGGTGGCGGAGCAGGAGGAGGCGACGACGTGGGAGCGGCCGACGAGGGAGGTGGAGACGCTGGAGCTGTTCCCGCTCAAAGCAGCCTGCTACGACCTGGAGCTGGAGGCGGACAGGTTCAGCCGATACGTGAGGGGCGGCGAGCAGCAGTGCAGGGAGATCTCCTTCTTCGACGTGGCCACCGGACGGGATCCGCCCCTGGAGCTCAGGCTCTGCAGCTTCGATCGGTATCTGGTCTAG |
