# Supplementary material for: Identification of the WUSCHEL-Related Homeobox (WOX) Gene Family, and Interaction and Functional Analysis of TaWOX9 and TaWUS in Wheat
Source: Int J Mol Sci. 2020 Feb 26;21(5):1581. doi: 10.3390/ijms21051581 (PMC7084607; doi:10.3390/ijms21051581)
Supplement: Supplementary file 1 [file ijms-21-01581-s001.zip › Supplementary Table S4.docx]

**Supplementary Table S4.** Primers used in the study.

Primers for Real-time PCR

| Primer name | Primer sequence |
| --- | --- |
| *TaSEP2F*  *TaSEP2R*  *TaLUXLF*  *TaLUXLR*  *TaPUB4F*  *TaPUB4R*  *TaBTB18F*  *TaBTB18R*  *TaFLXLF*  *TaFLXLR*  *TaFVEF*  *TaFVER*  *TaSEP3F*  *TaSEP3R*  *TaLUXLF*  *TaLUXLR*  *TaWUSF*  *TaWUSR*  *TaWOX2F*  *TaWOX2R*  *TaWOX3F*  *TaWOX3R*  *TaWOX4F*  *TaWOX4R*  *TaWOX5F*  *TaWOX5R*  *TaWOX6F*  *TaWOX6R*  *TaWOX7F*  *TaWOX7R*  *TaWOX8F*  *TaWOX8R*  *TaWOX9F*  *TaWOX9R*  *TaWOX11F*  *TaWOX11R*  *TaWOX12F*  *TaWOX12R*  *TaWOX13F*  *TaWOX13R*  *TaActinF*  *TaActinR* | 5’-CCCTCATCATCTTCTCCG-3’  5’-TTCAAGGTATTCAAGTCTGGTC-3’  5’-TCCATCATCGCCAGAGTG-3’  5’-TCGGGACCGAAGGAAAGC-3’  5’- CGAAGTGAGAACCGTAAA-3’  5’- TCAGCTCCAGGGATAGTG-3’  5’- TGCCGTGGCTGAAGCTGTG-3’  5’- TCGTGGAGTTGGCGGAGGAC-3’  5’-CTCCGTATTCGTGAGGTCTAT-3’  5’-TTCATGCCTGAGTTCTTGG-3’  5’-TCAATGAGGAAGCACGAT-3’  5’-CAACATCCCAAATAAGCA-3’  5’-CGCTCATCGTCTTCTCCA-3’  5’-TTATCAACCCGTGCCTTT-3’  5’ - TGTCAAGGTTTGGTGCTG -3’  5’ - GTCGTTTAGGCTGGTGGT -3’  5’- AAGCAGAGCGTCATGTGGAGA -3’  5’-ATGCCGCAGTCGTAGTAGAGGT-3’  5’- TCATCAGGGAGGAGGCGTAGT -3’  5’- AAGAGGTCCAGCGTCTTCAGC -3’  5’- AGCAGCAGATGGTGATGGA-3’  5’- GTGGAGGTGGAGCAAGAGG-3’  5’- CGTCAGGAACGCCACCAA -3’  5’- CGCCTCCAGCACCTTGATC -3’  5’-ACAGGCCACCATGTACCACCA-3’  5’-GGGAACAGGTTCAGGGTCTCG-3’  5’-CCACCATCGGTCTTCCATCC-3’  5’-GCTCCCAACGAATCCCATCT-3’  5’-GCCAAAGAAGAGGCGTATGAGG-3’  5’-CGAAGCAGGAGGAGGCAAAA-3’  5’-CAGGTATGAGGCTTGGTAATC-3’  5’-CTGAAATCTGTCCGTGCTG-3’  5’-CAGGTGAAGGTGCTGACGG-3’  5’-TCGTTGCTGCTGCTGTCG-3’  5’-ACAGCGGCATGGTGAACCC-3’  5’-AGCCCGAGCGACGTGGAAGA-3’  5’-TTGATCTCCTCAGGGCTTGACC-3’  5’-GGTGCGAGACTTGCGGTTC-3’  5’-AGCCTACTACTCGCAGATGCA-3’  5’-CTGTTGTTGGTCGAAGGAAAC-3’  5’-TGTTGTTCTCAGTGGAGGTTCT-3’  5’- CTGTATTTCCTTTCAGGTGGTG-3 |

Primers for plasmid construction

| Primer name | Primer sequence |
| --- | --- |
| *TaWUSBKF*  *TaWUSBKR*  *TaWUS-N-BKF*  *TaWUS-N-BKR*  *TaWUS-C-BKF*  *TaWUS-C-BKR*  *TaWOX9BKF*  *TaWOX9BKR*  *TaWOX9-N-BKF*  *TaWOX9-N-BKR*  *TaWOX9-C-BKF*  *TaWOX9-C-BKR*  *TaBTB18ADF*  *TaBTB18ADR*  *TaPUB4ADF*  *TaPUB4ADR*  *TaFLXL1ADF*  *TaFLXL1ADR*  *TaFVEADF*  *TaFVEADR*  *TaSEP3ADF*  *TaSEP3ADR*  *TaSEP2ADF*  *TaSEP2ADR*  *TaLUXLADF*  *TaLUXLADF*  *TaWUSBIFC-F*  *TaWUSBIFC-R*  *TaWOX9BIFCF*  *TaWOX9BIFCR*  *TaBTB18BIFCF*  *TaBTB18BIFCR*  *TaPUB4BIFCF*  *TaPUB4BIFCR*  *TaFLXL1BIFCF*  *TaFLXL1BIFCR*  *TaFVEBIFCF*  *TaFVEBIFCR*  *TaSEP3BIFCF*  *TaSEP3BIFCR*  *TaSEP2BIFCF*  *TaSEP2BIFCR*  *TaLUXBIFCF*  *TaLUXBIFCR*  *TaWUS-OE-F TaWUS-OE-R TaWOX9-OE-F TaWOX9-OE-R* | 5’- CATGGAGGCCGAATTCCATGGACAAGCAGAGCGTCAT-3’  5’-GGCCGCTGCAGGTCGACCATGTAAGGGGCGCGGAGAG-3’  5’CATGGAGGCCGAATTCCATGGACAAGCAGAGCGTCAT-3’  5’- GGCCGCTGCAGGTCGACGCCGAGGCGCTTCTTCTGGC-3’  5’-CATGGAGGCCGAATTCCGTCGACGTCAACGGCTCCCC-3’  5’-GGCCGCTGCAGGTCGACCATGTAAGGGGCGCGGAGAG-3’  5’-CATGGAGGCCGAATTCCATGGAGGCGCTGAGCGGGCG-3’  5’-GGCCGCTGCAGGTCGACGACCAGATACCGATCGAAGC-3’  5’-CATGGAGGCCGAATTCCATGGAGGCGCTGAGCGGGCG-3’  5’-GGCCGCTGCAGGTCGACGGCCTTGTGGTTCTGGAACC-3’  5’-CATGGAGGCCGAATTCCCGCGAGCGCCACCACCACAA-3’  5’-GGCCGCTGCAGGTCGACGACCAGATACCGATCGAAGC-3’  5’-CCATGGAGGCCAGTGAATTCATGCCGGAGGCACCTGCACG-3’  5’-AGCTCGAGCTCGATGGATCCGGCTCGGCAGCACTGAGAGG-3’  5’-CCATGGAGGCCAGTGAATTCATGGAAGATTTCTCACCGAG-3’  5’-AGCTCGAGCTCGATGGATCCTCTCCTCGCCGAGTTCCCAT-3’  5’-CCATGGAGGCCAGTGAATTCATGGCCGGTCGCCACCGTAA-3’  5’-AGCTCGAGCTCGATGGATCCTCTCCGTCCTGGAGCTCTTT-3’  5’-CCATGGAGGCCAGTGAATTCATGAAGGAGAGAGGCGGGTC-3’  5’-AGCTCGAGCTCGATGGATCCAGCCCTCGGAGTGCAGCTGG-3’  5’-CCATGGAGGCCAGTGAATTCATGGGGAGGGGGAGGGTGGA-3’  5’-AGCTCGAGCTCGATGGATCCAGGCAACCACGGGGGCATGA-3’  5’-CCATGGAGGCCAGTGAATTCATGGGTCGGGGGAAGGTGGA-3’  5’-AGCTCGAGCTCGATGGATCCTATCCAACCTGCAGATGATC-3’  5’-CCATGGAGGCCAGTGAATTCATGGAGGCGAGCAAGGGAGG-3’  5’-AGCTCGAGCTCGATGGATCCTATGGTGAGATCAAGAGTAC-3’  5’-CGAGCTCAAGCTTCGAAATGGACAAGCAGAGCGTCAT-3’  5’-CGACTGCAGAATTCGAACATGTAAGGGGCGCGGAGAG-3’  5’-CGAGCTCAAGCTTCGAAATGGAGGCGCTGAGCGGGCG-3’  5’-CGACTGCAGAATTCGAAGACCAGATACCGATCGAAGC-3’  5’-CGAGCTCAAGCTTCGAAATGCCGGAGGCACCTGCACG-3’  5’-CGACTGCAGAATTCGAAGGCTCGGCAGCACTGAGAGG-3’  5’-CGAGCTCAAGCTTCGAAATGGAAGATTTCTCACCGAG-3’  5’-CGACTGCAGAATTCGAATCTCCTCGCCGAGTTCCCAT-3’  5’-CGAGCTCAAGCTTCGAAATGGCCGGTCGCCACCGTAA-3’  5’-CGACTGCAGAATTCGAATCTCCGTCCTGGAGCTCTTT-3’  5’-CGAGCTCAAGCTTCGAAATGAAGGAGAGAGGCGGGTC-3’  5’-CGAGCTCAAGCTTCGAAAGCCCTCGGAGTGCAGCTGG-3’  5’-CGAGCTCAAGCTTCGAAATGGGGAGGGGGAGGGTGGA-3’  5’-CGAGCTCAAGCTTCGAAAGGCAACCACGGGGGCATGA-3’  5’-CGAGCTCAAGCTTCGAAATGGGTCGGGGGAAGGTGGA-3’  5’-CGAGCTCAAGCTTCGAATATCCAACCTGCAGATGATC-3’  5’-CGAGCTCAAGCTTCGAAATGGAGGCGAGCAAGGGAGG-3’  5’-CGAGCTCAAGCTTCGAATATGGTGAGATCAAGAGTAC-3’  5’-GGGGGACTCTTGACCATGGAGATGGACAAGCAGAGCGTCAT-3’  5’-TTCTTCTCCTTTACTAGTTCACATGTAAGGGGCGCGGAGAG-3’  5’-GGGGGACTCTTGACCATGGAGATGGAGGCGCTGAGCGGGCG-3’  5’-TTCTTCTCCTTTACTAGTTCAGACCAGATACCGATCGAAGC-3’ |
