# Supplementary material for: Identification of the WUSCHEL-Related Homeobox (WOX) Gene Family, and Interaction and Functional Analysis of TaWOX9 and TaWUS in Wheat
Source: Int J Mol Sci. 2020 Feb 26;21(5):1581. doi: 10.3390/ijms21051581 (PMC7084607; doi:10.3390/ijms21051581)
Supplement: Supplementary file 1 [file ijms-21-01581-s001.zip › Supplementary Table S1.docx]

| **Supplementary Table S1**: Summary of the *TaWOX* gene family | | | |
| --- | --- | --- | --- |
| **Name** | **Ensembl Gene Location** | **Amino acid Length** | **Ensemble Tanscript ID** |
| TaWUSa | 2A:724513458:724514647:1 | 308 | TraesCS2A02G491900.1 |
| TaWOX2a | 1A:33397501:33398955:-1 | 263 | TraesCS1A02G052000.1 |
| TaWOX2b | 1B:53364615:53365864:-1 | 264 | TraesCS1B02G069000.1 |
| TaWOX2d | 1D:35059826:35061088:-1 | 267 | TraesCS1D02G054000.1 |
| TaWOX3a | 5A:336949988:336951183:1 | 241 | TraesCS5A02G157300.1 |
| TaWOX3b | 5B:288891901:288893003:-1 | 241 | TraesCS5B02G156400.1 |
| TaWOX3d | 5D:254023305:254024410:1 | 242 | TraesCS5D02G162600.1 |
| TaWOX4a | 2A:738371677:738372966:1 | 232 | TraesCS2A02G514000.1 |
| TaWOX4b | 2B:740320190:740321561:-1 | 235 | TraesCS2B02G542600.1 |
| TaWOX4d | 2D:606709221:606710431:1 | 235 | TraesCS2D02G515600.1 |
| TaWOX5a | 5A:111588730:111590895:1 | 320 | TraesCS5A02G085000.1 |
| TaWOX5b | 5B:118451983:118454221:1 | 323 | TraesCS5B02G091000.1 |
| TaWOX5d | 5D:108103399:108105722:1 | 324 | TraesCS5D02G097400.1 |
| TaWOX6a | 4A: 170,708,103-170,711,065 | 307 | TraesCS4A02G130200.1 |
| TaWOX6b | 4B: 382,691,977-382,694,806 | 309 | TraesCS4B02G174400.1 |
| TaWOX6d | 4D: 306,795,298-306,798,208 | 306 | TraesCS4D02G176400.1 |
| TaWOX7a | 3A:465225214:465228773:1 | 523 | TraesCS3A02G247200.1 |
| TaWOX7b | 3B:438378936:438382259:-1 | 523 | TraesCS3B02G272200.1 |
| TaWOX7d | 3D:339473290:339476679:-1 | 521 | TraesCS3D02G244300.1 |
| TaWOX8a | 3A:588932808:588937056:1 | 265 | TraesCS3A02G341700.1 |
| TaWOX8b | 3B:586694870:586698391:1 | 261 | TraesCS3B02G373800.1 |
| TaWOX8d | 3D:447560283:447562999:1 | 263 | TraesCS3D02G335500.1 |
| TaWOX9a | 3A:617060395:617061453:-1 | 210 | TraesCS3A02G368100.1 |
| TaWOX9b | 3B:631036656:631037718:-1 | 208 | TraesCS3B02G399800.1 |
| TaWOX9d | 3D:474614857:474615873:-1 | 208 | TraesCS3D02G361100.1 |
| TaWOX10a | 3A:45776166:45777448:1 | 260 | TraesCS3A02G073500.1 |
| TaWOX10b | 3B:56055903:56057760:1 | 261 | TraesCS3B02G087800.1 |
| TaWOX10d | 3D:33294918:33295992:1 | 261 | TraesCS3D02G073300.1 |
| TaWOX11a | 2A:53782606:53785288:1 | 265 | TraesCS2A02G100700.1 |
| TaWOX11b | 2B:81755546:81758516:1 | 261 | TraesCS2B02G117900.1 |
| TaWOX11d | 2D:52227203:52229885:1 | 264 | TraesCS2D02G100200.1 |
| TaWOX12a | 1A:563818671:563823103:1 | 494 | TraesCS1A02G399400.1 |
| TaWOX12b | 1B:652781930:652786496:1 | 493 | TraesCS1B02G427400.1 |
| TaWOX12d | 1D:470219711:470224514:1 | 494 | TraesCS1D02G406900.1 |
| TaWOX13a | 3A:606444775:606446830:-1 | 303 | TraesCS3A02G358100.1 |
| TaWOX13b | 3B:616425121:616426978:-1 | 299 | TraesCS3B02G391100.1 |
| TaWOX13d | 3D:463197196:463199275:-1 | 298 | TraesCS3D02G352500.1 |
| TaWOX14a.1 | 3A:606515981:606519197:-1 | 288 | TraesCS3A02G358200.1 |
| TaWOX14a.2 | 3A:606573438:606576220:-1 | 290 | TraesCS3A02G358400.1 |
| TaWOX14b | 3B:616645332:616647892:-1 | 290 | TraesCS3B02G391200.1 |
| TaWOX14d.1 | 3D:463227796:463230501:-1 | 285 | TraesCS3D02G352600.1 |
| TaWOX14d.2 | 3D:463378560:463381808:-1 | 291 | TraesCS3D02G352700.1 |
