# Supplementary material for: Identification of the WUSCHEL-Related Homeobox (WOX) Gene Family, and Interaction and Functional Analysis of TaWOX9 and TaWUS in Wheat
Source: Int J Mol Sci. 2020 Feb 26;21(5):1581. doi: 10.3390/ijms21051581 (PMC7084607; doi:10.3390/ijms21051581)
Supplement: Supplementary file 1 [file ijms-21-01581-s001.zip › Supplementary Table S2.docx]

**Supplementary Table S2**: Summary of the AtWOX and OsWOX gene family

| **Name** | **Ensemble Tanscript ID** |
| --- | --- |
| OsWOX1 | Os04g0663600 |
| OsWOX2 | Os05g0118700 |
| OsWOX3 | Os12g0101600 |
| OsWOX4 | Os04g0649400 |
| OsWOX5 | Os01g0840300 |
| OsWOX6 | Os03g0325600 |
| OsWOX7 | Os01g0667400 |
| OsWOX8 | Os01g0818400 |
| OsWOX9 | Os01g0854500 |
| OsWOX10 | Os08g0242400 |
| OsWOX11 | Os07g0684900 |
| OsWOX12 | Os05g0564500 |
| OsWOX13 | Os01g0667400 |
| AtWUSCHEL | AT2G17950 |
| AtWOX1 | AT3G18010 |
| AtWOX2 | AT5G59340 |
| AtWOX3 | AT2G28610 |
| AtWOX4 | AT1G46480 |
| AtWOX5 | AT3G11260 |
| AtWOX6 | AT2G01500 |
| AtWOX7 | AT5G05770 |
| AtWOX8 | AT5G45980 |
| AtWOX9 | AT2G33880 |
| AtWOX10 | AT1G20710 |
| AtWOX11 | AT3G03660 |
| AtWOX12 | AT5G17810 |
| AtWOX13 | AT4G35550 |
| AtWOX14 | AT1G20700 |
