# Supplementary figures and images for: Identification of the WUSCHEL-Related Homeobox (WOX) Gene Family, and Interaction and Functional Analysis of TaWOX9 and TaWUS in Wheat
Source: Int J Mol Sci. 2020 Feb 26;21(5):1581. doi: 10.3390/ijms21051581 (PMC7084607; doi:10.3390/ijms21051581)

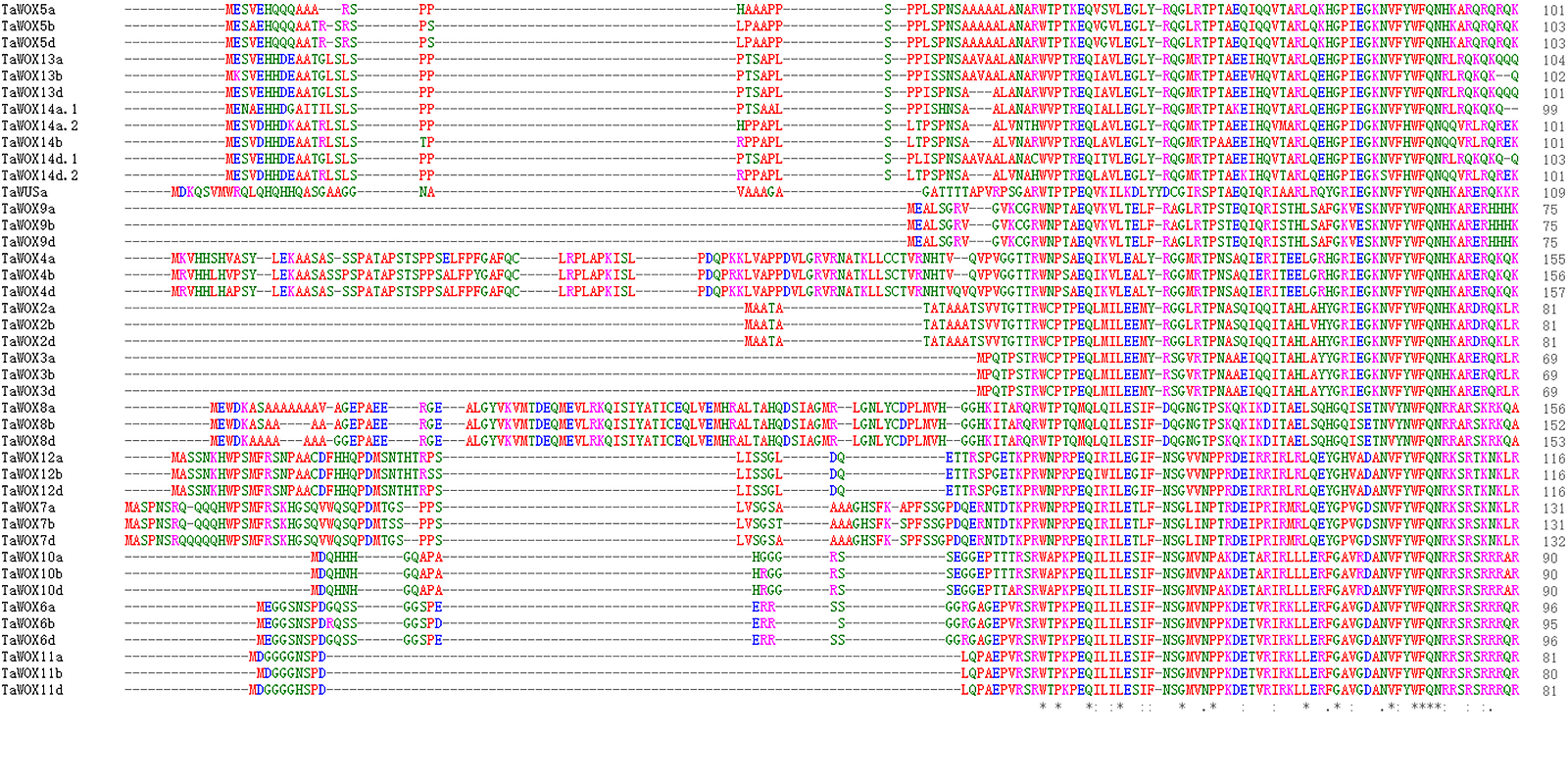

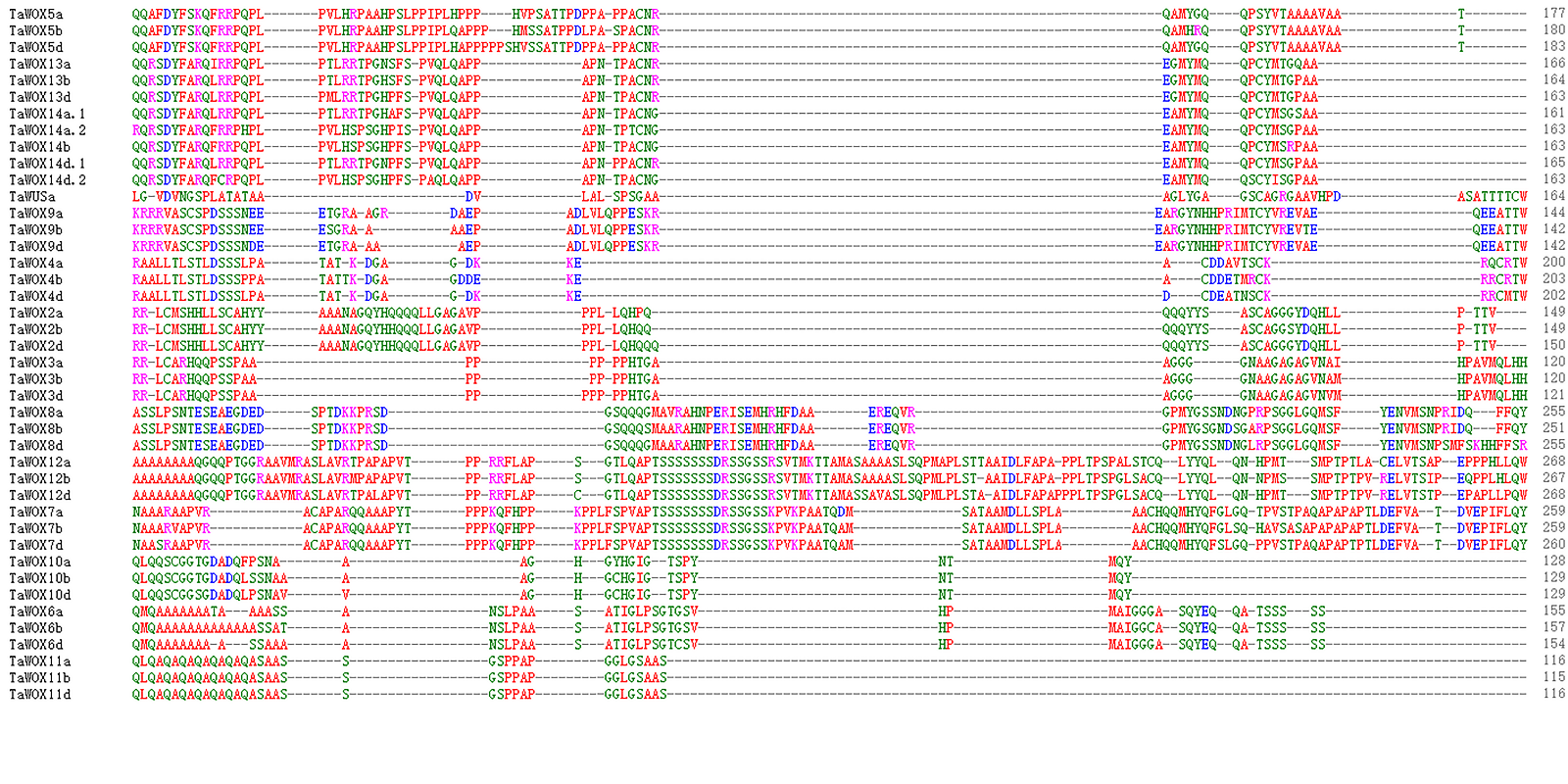

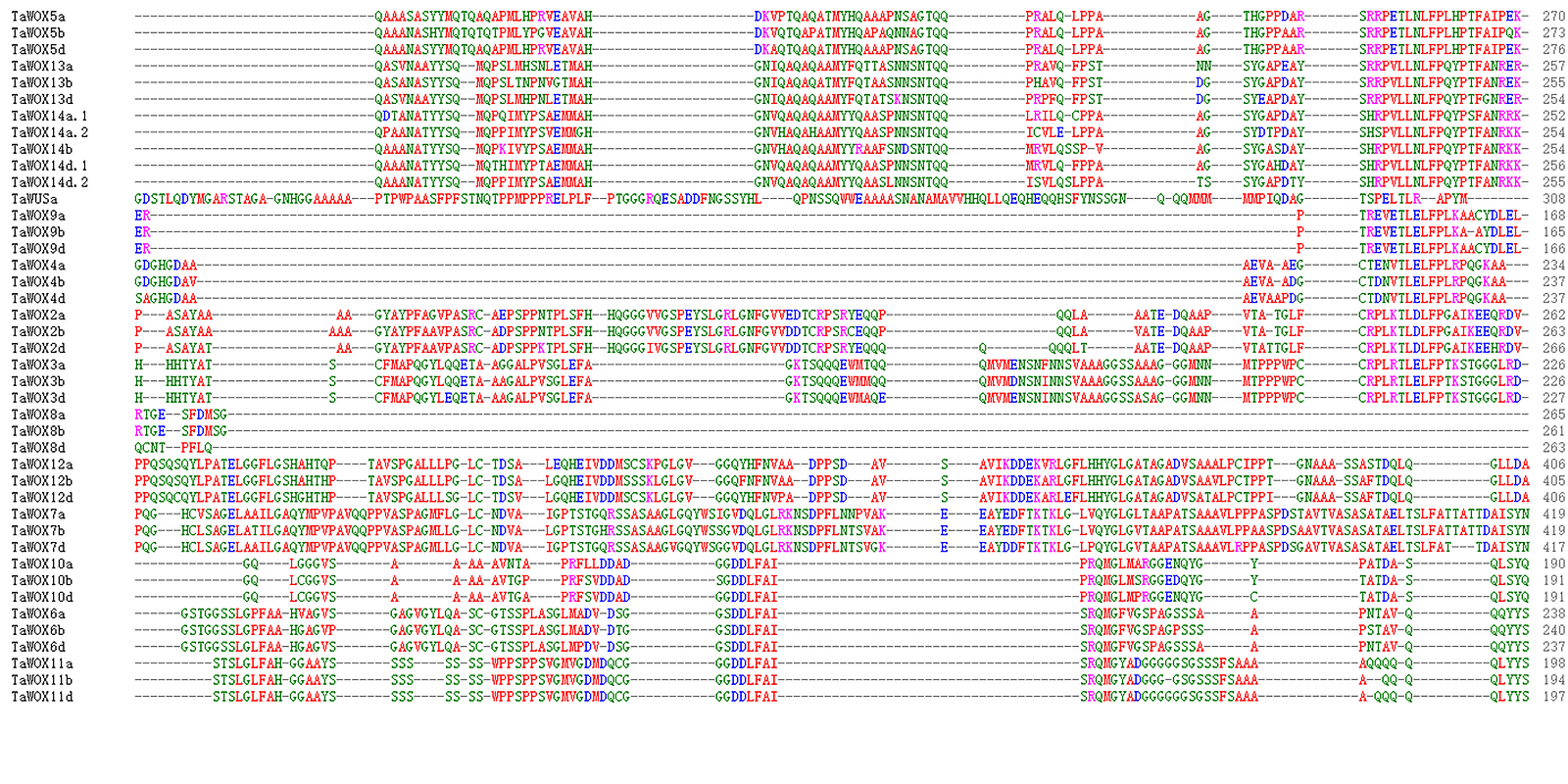

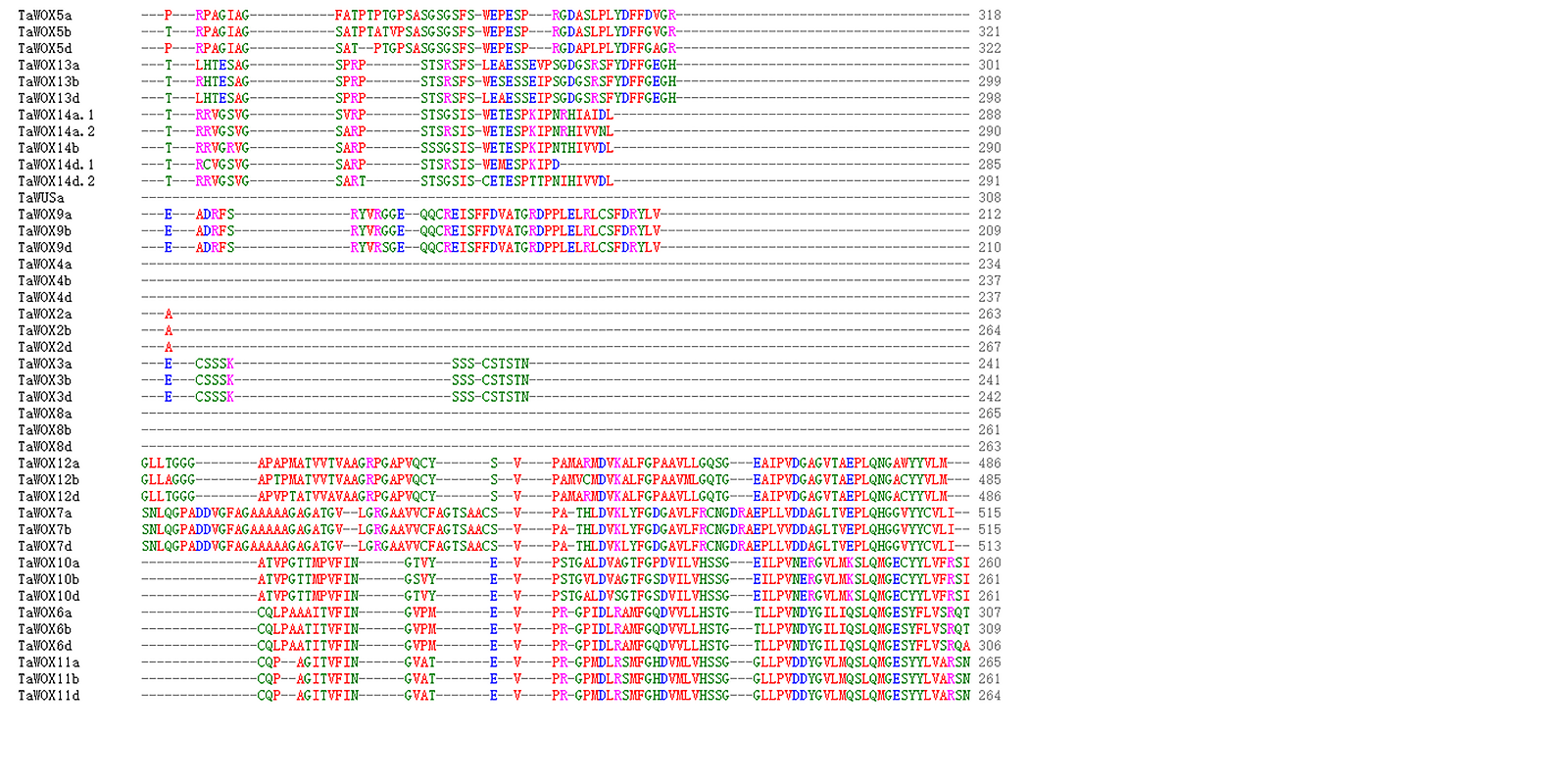


**Supplementary Figure S1.** Multiple sequence alignment of all identified TaWOX proteins.

Supplement: Supplementary file 1 [file ijms-21-01581-s001.zip › Supplementary Figure S1.docx]
