# Supplementary material for: Identification of the WUSCHEL-Related Homeobox (WOX) Gene Family, and Interaction and Functional Analysis of TaWOX9 and TaWUS in Wheat
Source: Int J Mol Sci. 2020 Feb 26;21(5):1581. doi: 10.3390/ijms21051581 (PMC7084607; doi:10.3390/ijms21051581)
Supplement: Supplementary file 1 [file ijms-21-01581-s001.zip › Supplementary Figure S2-S6.doc]

**C**

**D**

**E**

**F**

**Supplementary Figure S2.** The phylogenetic tree analysis of interacting proteins of TaWUS and TaWOX9. (**A**) The phylogenetic tree of TaFLXL1 and its homologs. (**B**) TaFVE and its homologs. (**C**) TaPUB4 and its homologs. (**D**) TaSEP2, TaSEP3 and their homologs. (**E**) TaBTB18 and its homologs. (**F**) TaLUXL and its homologs. Phylogenetic trees were constructed with the program MEGA X (https://www.megasoftware.net/download_form) using the Neighbor-joining approach with 1000 bootstrap replicates. TaWOX9 was selected as a out-group protein.

**A**

**B**

**Supplementary Figure S3.** Further interaction analysis of PUB4 protein and WOX family proteins in different plants. (**A**) Interaction of AtPUB4 and AtWUS and interaction of OsPUB4 and OsWOX1 were detected in yeast cells. (**B**) Interaction of TaPUB4 and TaWOX3 and interaction of TaPUB4 and TaWOX8 were detected in yeast cells.

**Supplementary Figure S4.** Expression profiles of seven interacting protein genes and *TaWOX14* and *TaWOX10* in different tissues or organs. The tissues or organs evaluated were the leaf, leaf sheath, stem, root, glume, spike (young spike), ovary, anther, and grain from left to right. Vs, vegetative stage. Rs, reproductive stage. Each value represents the mean of three biological replicates ± SE. *TaActin* was chosen as an internal control. The primers used are listed in Supplementary Table S4. Expression profiles of *TaWOX14* and *TaWOX10* were generated from the expVIP database (http://www.wheat-expression.com/).

**Supplementary Figure S5.** Further analysis of TaWUS overexpression (TaWUS-OE) in *Arabidopsis*. (**A**) Quantitative real time polymerase chain reaction (RT-qPCR) identification of transgenic TaWUS-OE and TaWOX9 *Arabidopsis* plants. *AtActin* was used as a reference gene. (**B**) 30-day-old plants (TaWUS-OE and WT). **(C-D**) The siliques were from the 40-day-old plants. The phenotypes shown represent the phenotypes of two independent transgenic lines. (**E**) 40-day-old plants (TaWUS-OE and WT). ((**F-G**) Phenotype of branch nodes of WT (**F**) and TaWUS-OE (**G**). Scale bars = 3cm (**B** and **E**), 0.5 cm ( **C** and **D**). The above phenotypes were derived from two independent transgenic lines.

**A**

**B**

| **Gene name** | **Orthologs in Arabidopsis** | **Description** | **Biological Process** |
| --- | --- | --- | --- |
| **TaPUB4** | **AtPUB4** | **PUB4 encodes a functional ubiquitin-protein ligase.** | **anther wall tapetum development, cellular response to reactive oxygen species, root development** |
| **TaLUX** | **AT2G38300.1** | **myb-like HTH transcriptional regulator family protein** | **regulation of transcription, DNA-templated** |
| **TaFVE** | **AtFVE** | **Controls flowering.** | **cell differentiation, chromatin organization, flower development** |
| **TaBTB18** | **AtFVE** | **Encodes a protein (BT2) that is an essential component of the TAC1-mediated telomerase activation pathway.** | **auxin-activated signaling pathway, embryo development, pollen development** |
| **TaSEP2** | **AtSEP2 or AtAGL4** | **MADS-box protein, binds K domain of AG in vivo** | **flower development, specification of floral organ identity** |
| **TaSEP3** | **AtSEP3** | **Member of the MADs box transcription factor family. SEP3 is redundant with SEP1 and 2.** | **cell differentiation, flower development** |
| **TaFLX** | **AT3G14750.1** | **structural maintenance of chromosomes domain protein** | **cell differentiation, flower development** |

**Supplementary Figure S6. (A)** Predicting the functions of interacting proteins of TaWOX9 and TaWUS by STRING interaction analysis. The interacting proteins were mapped to Arabidopsis thaliana homologs by searching the STRING 11.0 database (http://string-db.org) with a confidence cutoff of 0.4. (**B**) Information on the interacting proteins of TaWUS and TaWOX9 identified by yeast two-hybrid and their orthologs in *Arabidopsis*.
